# Low Levels of Zinc Exchanged into Cu‐SSZ‐13 Increase Methanol Production in the Partial Oxidation of Methane to Methanol

**DOI:** 10.1002/open.202500352

**Published:** 2025-07-29

**Authors:** Motunrayo Ogunleye, Hridita Purba Saha, Ayman M. Karim, Daniel F. Shantz

**Affiliations:** ^1^ Department of Chemical and Biomolecular Engineering Tulane University 6823 St. Charles Avenue New Orleans LA 70118 USA; ^2^ Department of Chemical Engineering Virginia Polytechnic Institute and State University Blacksburg VA 24061 USA

**Keywords:** copper, methane partial oxidation, methanol, SSZ‐13, zinc

## Abstract

Cu‐SSZ‐13 is currently used industrially for the selective catalytic reduction of nitric oxides and has shown potential for the direct oxidation of methane to methanol. Here, how exchanging a small amount of zinc into Cu‐SSZ‐13 impacts the methane to methanol activity is reported. Samples containing only zinc are catalytically inactive. By contrast, small levels of zinc (Zn/Al = 0.06 or less) lead to a marked increase in methanol production. In the best case, Cu,Zn‐SSZ‐13 (Cu/Al = 0.21, Zn/Al = 0.06) has a site time yield (STY) of 26.4 ± 0.42 mmol mol‐Cu‐h^−1^ and specific activity (SA) of 11.5 ± 0.18 μmol g‐h^−1^. This is over an 80% increase in both STY and SA over Cu‐SSZ‐13 samples with comparable copper loadings (Cu/Al = 0.2–0.26). A similar, but slightly less significant improvement, is observed for Cu,Zn samples at a copper loading of ≈0.12, where 50% increase in methanol production over just copper samples is observed. Infrared spectroscopy results suggest that the presence of zinc makes the copper more electron‐deficient, providing one possible explanation for the increased activity.

## Graphical abstract

A little zinc helps! The addition of small quantities of zinc to Cu‐SSZ‐13 leads to a significant enhancement in methanol production in the partial oxidation of methane to methanol.

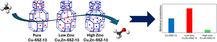

## 1 Introduction

Methane, an abundant hydrocarbon which has a high energy density, is primarily used as a fuel source for the generation of electricity, as fuel to power vehicles, cookers, and as a key raw material for the production of industrial chemicals.^[^ ^1^, ^2^, ^3^ ^]^ However, methane is a powerful greenhouse gas (28 times more potent than carbon dioxide), and there is a global push for a shift away from methane as a fuel to renewable energy sources.^[^ ^4^, ^5^ ^]^ The International Energy Agency, IEA, estimates that the annual emission of methane is 580 million tons,^[^ ^6^ ^]^ with 60% of the emission coming from human activity. Further, 140 billion cubic meters (bcm) of natural gas is flared globally each year due to challenges around its transport (high cost of building and maintaining a gas pipeline, long distance between offshore oil fields and possible point of methane use, lack of storage facilities, etc.). This flaring leads to the emission of carbon dioxide, which is also a greenhouse gas, in the case of complete combustion, and in some cases, flaring leads to the emission of soot, carbon dioxide, and carbon monoxide (CO). In addition to being harmful to the environment, these pollutants are also harmful to human health.^[^ ^7^, ^8^, ^9^ ^]^ Methane has industrial applications as it is an important raw material for the production of fertilizers, methanol, and a source for hydrogen in the oil and gas industry.^[^ ^10^, ^11^, ^12^, ^13^, ^14^ ^]^ However, methane has to be converted to synthetic gas first through a process called steam reforming before it can be used for most of its industrial applications.^[^ ^15^, ^16^ ^]^

The direct partial oxidation of methane to methanol, which is one route to enable more utilization of methane, is regarded as a grand challenge problem.^[^ ^17^ ^]^ Methanol is a liquid fuel that is easy to handle, transport, and store. Methanol has numerous industrial applications and can also be upgraded into a range of other molecules, including olefins, alkanes, and aromatics (i.e., gasoline).^[^ ^18^, ^19^, ^20^, ^21^, ^22^, ^23^ ^]^ Many materials have been studied as catalysts for this reaction, including metal oxides,^[^ ^24^ ^]^ metal organic frameworks,^[^ ^25^, ^26^ ^]^ and zeolites.^[^ ^27^, ^28^, ^29^, ^30^ ^]^ SSZ‐13 is an aluminosilicate zeolite material with the chabazite topology that is used commercially in the selective catalytic reduction of NO,^[^ ^31^, ^32^ ^]^ and compositional variants (e.g., SAPO‐34) are used as a catalyst for methanol to olefins.^[^ ^18^, ^20^, ^33^ ^]^ It has also been shown to be a potential catalyst for partial oxidation of methane to methanol at moderate temperature (200 to 250 °C). Transition metals such as copper and iron are ion‐exchanged into zeolites and are the active centers for the chemistry.^[^ ^34^, ^35^ ^]^ Prior works in our lab and other labs have shown copper‐exchanged SSZ‐13 (Cu‐SSZ‐13) and copper‐exchanged SSZ‐39 (Cu‐SSZ‐39) to be good catalysts for partial oxidation of methane to methanol using different oxidants such as N_2_O, water, and O_2_, both in the gas phase.^[^ ^36^, ^37^, ^38^, ^39^, ^40^, ^41^, ^42^, ^43^, ^44^ ^]^ There are two key factors that make Cu‐SSZ‐13 a viable catalyst for the methane to methanol reaction. The first is the presence of copper cations, and the second is the presence of Brønsted acid sites (for increased stability of methanol formed and prevention of overoxidation to carbon dioxide).^[^ ^45^, ^46^ ^]^

Zeolites ion‐exchanged with two different metals have been used for different reactions^[^ ^47^, ^48^, ^49^ ^]^ and have been shown sometimes to give an improvement in catalytic activity compared to zeolites exchanged with just one of the two metals. As an example, iso‐butane cracking over zinc oxide‐doped platinum‐ZSM‐5 in the ratio Zn:Pt = 1:10 has improved yields compared to just Pt and other Zn–Pt ratios.^[^ ^48^, ^50^ ^]^ This is part of the motivation for the work described here. A theme that emerges in the literature is that the best catalyst performance is observed when the second metal is exchanged into the zeolite at a lower loading^[^ ^49^ ^]^ compared to the metal loading of the single metal exchanged zeolite. The literature hypothesis is that the second metal acts as a support or promoter and is not the active metal. Hence, exchanging more of the second metal leads to a reduction in activity. Here, we exchanged zinc into Cu‐SSZ‐13 to increase copper activity for methane to methanol conversion in Cu‐SSZ‐13. Cu‐SSZ‐13 was chosen as it is possible to easily control the loading of the zinc into the zeolite. We report our findings on how the ion‐exchange protocol, copper loading, copper to zinc ratio, and sequence of metal exchange impact the production of methanol as compared to Cu‐SSZ‐13.

## 2 Experimental Section

### 2.1 Materials

N,N,N‐trimethyl‐1‐adamantylammonium hydroxide (TMAdaOH, 20 wt%) was purchased from SACHEM. 5.0 N Sodium hydroxide solution was purchased from VWR. Aluminum hydroxide powder and Ludox HS‐40 were purchased from Sigma‐Aldrich. Ammonium nitrate (95% min.) was purchased from Beantown Chemicals. Copper(II) sulfate pentahydrate (ACS, 98%–102%) was purchased from Alfa Aesar. Zinc(II) sulfate heptahydrate (99–103% ACS grade) was purchased from Thermo Fischer Scientific.

### 2.2 Zeolite Synthesis

SSZ‐13 was made from a gel of composition 1 SiO_2_: 0.133 Al_2_O_3_: 0.25 TMAdaOH: 0.25 Na_2_O: 44 H_2_O using the procedure from Di Iorio and coworkers.^[^ ^51^ ^]^ As an example, SSZ‐13 was made by mixing 11.08 g of N,N,N‐trimethyl‐1‐adamantylammonium hydroxide and 5.875 g of deionized water (18 MΩ) together in a beaker for 15 min at room temperature. Then, 1.284 g of 5.0 M sodium hydroxide solution and 0.109 g of aluminum hydroxide powder were added and stirred for another 15 min at room temperature. Finally, 3.15 g of Ludox^®^ HS‐40 colloidal silica was added and stirred for two hours at room temperature. The mixture was then transferred into two Teflon‐lined Parr acid digestion bombs and loaded into the rotating rack of an oven for six days at a temperature set at 160 °C and rotated at ≈60 rpm. After six days, the samples were cooled, and the solids were collected by vacuum filtration and then washed with excess deionized water and dried at 80 °C overnight in an oven. The dried as‐made SSZ‐13 was calcined in an electric furnace at 550 °C for 8 h to remove the organic structure‐directing agent from the pores.

### 2.3 Ion Exchange Protocol

After calcination, all samples were converted to the ammonium form by ion exchange, 1 g of zeolite with 250 mL of 0.1 M aqueous solution of ammonium nitrate at 80 °C for 8 h. The solids were recovered by vacuum filtration, washed with deionized water, and dried at room temperature overnight. The NH_4_‐SSZ‐13 was used to make the mixed metal (X,Y‐SSZ‐13) catalysts. X,Y‐SSZ‐13 denotes the mixed metal catalysts where X stands for the first metal exchanged and Y stands for the second metal exchanged into the zeolite sample.

### 2.4 Cu‐SSZ‐13 Exchange

Copper‐exchanged SSZ‐13 (Cu‐SSZ‐13) samples were prepared by ion exchange of 1 g of NH_4_‐SSZ‐13 with 100–300 mL of a 0.05–0.1 M copper (II) sulfate solution at room temperature for 1 h. The product was recovered by vacuum filtration and washed with deionized water. The wet solids were placed in the oven at 80 °C overnight to dry.

### 2.5 Zn‐SSZ‐13 Exchange

Zinc‐exchanged SSZ‐13 (Zn‐SSZ‐13) samples were prepared by ion exchange of 1 g of NH_4_‐SSZ‐13 with 100–500 mL of a 0.05–0.1 M zinc (II) sulfate (ZnSO_4_) at 80 °C for 30 min to 2 h. The product was recovered by vacuum filtration and washed with deionized water. The wet solids were placed in the oven at 80 °C overnight to dry.

### 2.6 Cu,Zn‐SSZ‐13 Exchange

Copper, zinc‐exchanged SSZ‐13(Cu,Zn‐SSZ‐13) samples were prepared by first ion exchanging 1 g of NH_4_‐SSZ‐13 with 100–300 mL of 0.05–0.1 M copper sulfate solution at room temperature for 1 h to obtain Cu‐SSZ‐13. The product was recovered by vacuum filtration and washed with excess deionized water. The wet solids were placed in the oven at 80 °C overnight to dry. The Cu‐SSZ‐13 samples were then further ion exchanged by mixing 1 g of Cu‐SSZ‐13 with 100–300 mL of 0.001–0.05 M ZnSO_4_, depending on the copper loading, at room temperature or at 50–80 °C for 1 h to obtain Cu,Zn‐SSZ‐13 samples. The wet solids (Cu,Zn‐SSZ‐13) were placed in the oven at 80 °C overnight until dry.

### 2.7 Zn,Cu‐SSZ‐13 Exchange

Zinc, copper‐exchanged SSZ‐13(Zn,Cu‐SSZ‐13) samples were prepared by first ion exchanging 1 g of NH_4_‐SSZ‐13 with 200 mL of a 0.05 M zinc sulfate at 80 °C for 1 h to obtain Zn‐SSZ‐13. The product was recovered by vacuum filtration and washed with deionized water. The wet solids were placed in the oven at 80 °C overnight to dry. The Zn,Cu‐SSZ‐13 samples were obtained by mixing 1 g of Zn‐SSZ‐13 with 100–300 mL of a 0.05–0.1 M copper (II) sulfate solution at room temperature for 1 h. The wet solids (Zn,Cu‐SSZ‐13) were placed in the oven at 80 °C overnight until dry.

### 2.8 Analytical

X‐ray diffraction (XRD) measurements were carried out using a Rigaku MiniFlex 600 diffractometer with CuKα radiation at a speed of 0.25 deg(°) min^−1^ and a step size of 0.02 deg(°)/step over the 2θ range of 4°–60°. The particle size and morphology of the SSZ‐13 samples were determined using field‐emission scanning electron microscopy with a Hitachi 4800 high‐resolution scanning electron microscope with a voltage set at 20 kV. The elemental composition of the samples was determined by use of energy‐dispersive X‐ray spectroscopy (EDS) attached to a Hitachi S‐3400 scanning electron microscope, running at 20 kV, equipped with an Oxford Instruments EDS detector.

### 2.9 Diffuse Reflectance Infrared Fourier Transform Spectroscopy (DRIFTS)

DRIFTS was used to characterize the interaction of the Cu‐SSZ‐13 samples with CO. The in situ DRIFTS experiments were performed using a Thermo Scientific IS‐50R FT‐IR equipped with an MCT/A detector. A spectral resolution of 4 cm^−1^ was used to collect spectra, which are reported in the Kubelka–Munk (KM) units. A ≈50 mg of the sample (particle size: 25–90 μm) was loaded into a Harrick Praying Mantis high‐temperature DRIFTS reaction chamber. The chamber was sealed and connected to a gas flow system with temperature control, and all measurements were conducted under atmospheric pressure. Each spectrum represents the average of 32 scans. The Cu, Zn‐SSZ‐13 samples underwent in situ pretreatment in 2% O_2_ (99.999%, Airgas) in N_2_ at 550 °C for 30 min, followed by cooling to 35 °C in pure N_2_. Since the samples were purged and cooled in N_2_, it is anticipated that the autoreduction of Cu(II) to Cu(I) will occur, as it was observed in other studies.^[^ ^52^ ^]^

### 2.10 Catalytic Testing

Methane (≥99.99%, O_2_ ≤ 5 ppm v/v), helium (≥99.999%, O_2_ ≤ 1 ppm v/v), 1% oxygen in nitrogen (1.000%, concentration mole% O_2_ 1.005%, balance N_2_), and air ((O_2_ = 20%‐22%, H_2_O ≤ 2 ppm v/v) cylinders were obtained from Airgas. The mass flow controllers (MFCs) for methane, helium, and air streams were purchased from Aalborg Instruments. The MFC for the 1% oxygen in nitrogen stream was purchased from Bronkhorst. Deionized water was vaporized using a Perma Pure MH‐Series humidifier. The water line to the reactor was wrapped with heat tape and temperature controlled with a heater from Ace Glass, with the temperature set to 70 °C. The product line after the reactor was also heat wrapped with the temperature set to 80 °C. Elimination of trace oxygen in the methane and helium streams was achieved by placing oxygen traps from Agilent Technologies on the methane and helium lines. Analysis of the product stream from the reactor was performed using a gas chromatograph from Agilent Technologies model 7890N, equipped with a HP‐Plot‐Q column (Agilent, 30 m, 0.32 mm, 19091 P‐Q04), flame ionization detector for methane and methanol monitoring, and thermal conductivity detector (TCD) for methane, carbon dioxide, and CO monitoring.

**Figure 1:**
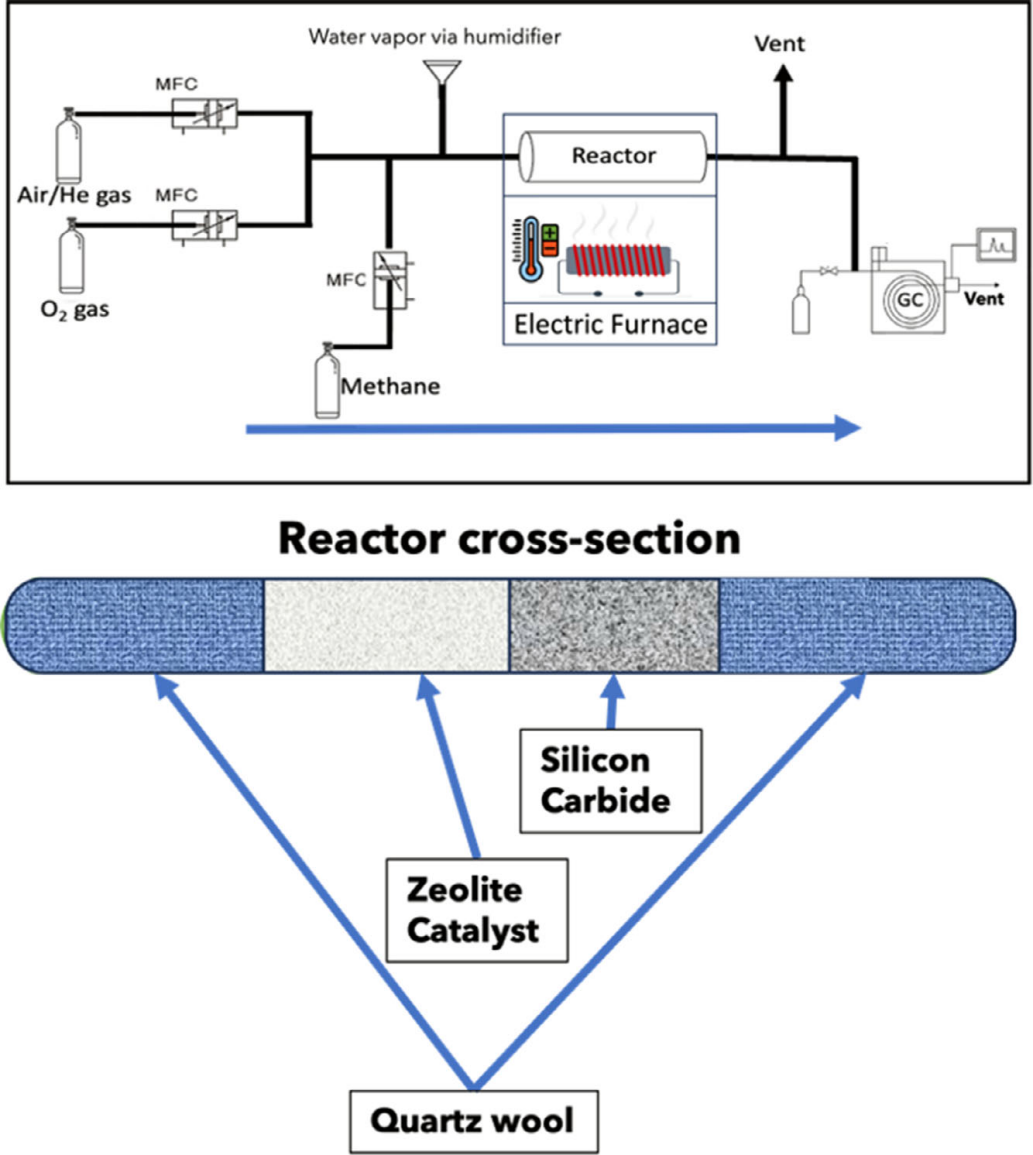
Reactor setup for the reaction and cross‐section of the reactor tube.

The samples are pelletized by first compressing the dry powder form of the samples using a benchtop hand press into a solid mass. The solid mass was then sieved through two sieves (mesh size of 500 and 850 μm) to obtain particles 500–850 μm in diameter. The reactor is a quarter‐inch diameter stainless‐steel tube that is nine inches in length. The reactor bed was packed by loading one end with quartz wool (9–30 micron, Thermo Fisher Scientific), followed by 800 mg of silicon carbide (46 grit, ThermoFisher Scientific) to provide support for the catalyst. As a control, the reaction was run with the reactor tube loaded with silicon carbide and quartz wool, no catalyst, and there was no methanol production observed. 400 mg of pelletized catalyst sample was then loaded into the reactor tube, and then the reactor tube was then sealed on the other end with quartz wool. The reactor tube was then placed in an electric furnace and connected to the reactant lines and its exit leading to the gas chromatograph as shown in **Figure** **1**. The reaction was performed by first activating the catalyst by heating the reactor to 550 °C for 2 h while flowing air at 50 mL min^−1^. The air was turned off, and the activated catalyst was then purged for 30 min using helium at 40 mL min^−1^ while cooling the reactor to the reaction temperature, 25 °C. The next step after purging was flowing just helium and methane over the catalyst at the reaction temperature for 40 min (20 mL min^−1^ for each gas). Then the remaining gases (oxygen in N_2_ flowing at 1mL/min and water vapor via the humidifier) were turned on, with methane and helium still flowing at 20mL/min each, which is referred to as t = 0. Readings were taken on the gas chromatograph every 20 min and a steady‐state methanol production was observed typically after 150–200 min of reaction time consistent with prior work by Narsimhan et al.^[^ ^36^ ^]^ The weight hourly space velocity is 6000 mL h^−1^ gcat^−1^ with the total reactant flow rate held at 40 mL min^−1^ (50% CH_4_, 2% water, 250 ppm O_2_, balance He). Two parameters used to quantify the catalytic activity are the specific activity (SA) and the site time yield (STY). The SA is defined as the catalytic activity per gram of catalyst (units of *μ*mol g‐h^−1^)

$$\text{Specific Activity =}\frac{\text{amount of methanol produced per hour}}{\text{total mass of catayst}}$$

The STY is the measured reaction rate normalized by the number of active sites. Here, we assume all copper is active, i.e., the total moles of copper are used in normalization (units of mmol mmol^−1^ Cu‐h)

$$\text{Site Time Yield= }\frac{\text{amount of methanol produced per hour}}{\text{total mole of Copper in catalyst}}$$

## 3 Results and Discussion

**Figure 2:**
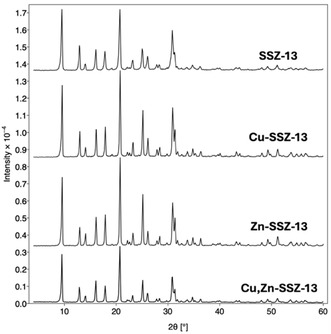
XRD data for SSZ‐13, Cu‐SSZ‐13, Zn‐SSZ‐13, and Cu,Zn‐SSZ‐13 (from top to bottom).

The SSZ‐13 samples as‐made, Cu‐SSZ‐13, Zn‐SSZ‐13, Cu,Zn‐SSZ‐13, and Zn,Cu‐SSZ‐13 were characterized using powder X‐ray diffraction (PXRD) to determine phase purity. **Figure** **2** shows the PXRD for the samples studied. The PXRD patterns show no discernable differences in the number and location of the peaks across all the samples. This indicates that ion exchange did not lead to the formation of metal clusters or metal oxide clusters large enough to diffract X‐rays. PXRD measurement of the samples was indexed against the simulated diffraction pattern of the standard hexagonal SSZ‐13 framework. The values of lattice constants were determined to be *a* = *b* = 13.629 ± 0.002, *c* = 14.848 ± 0.004 within the 99% confidence interval. The XRD patterns of the samples were measured after use as a catalyst in the methane to methanol reactor, and the patterns showed no changes (Figure S2, Supporting Information).

Scanning electron microscope (SEM) images of the Cu‐SSZ‐13, Zn‐SSZ‐13, and Cu,Zn‐SSZ‐13 (Figure S1, Supporting Information) did not show any difference when compared to the parent as‐synthesized SSZ‐13. The observed particle shape of the four SSZ‐13 samples from SEM images is consistently rhombohedral. The average particle size of the samples also remains unchanged regardless of ion exchange protocols at ≈5 μm. This shows that there was no change to the physical and surface structure of the samples because of copper or zinc ion exchange. The amount of metal exchanged into the samples was controlled by varying the volume of metal salt solution, duration of exchange, or the temperature of exchange, and quantified using the EDS analysis.

EDS analysis of the elemental composition of the SSZ‐13 gives an Si/Al of 6.62 ± 0.62. Cu‐SSZ‐13 (Cu‐SSZ‐13) samples, with Cu/Al values of 0.1–0.26, were prepared by controlling one or a combination of copper concentration, volume of copper sulfate solution, and temperature of ion exchange. Zinc loading into NH_4_‐SSZ‐13 and Cu‐SSZ‐13 was also varied in a similar manner using concentration, volume of metal solution, and temperature of ion exchange. Zinc loading was determined to vary between 0.01 and 0.24. Details can be found in Supporting Information (Table S1, Supporting Information).

### 3.1 Catalytic Testing

**Figure 3:**
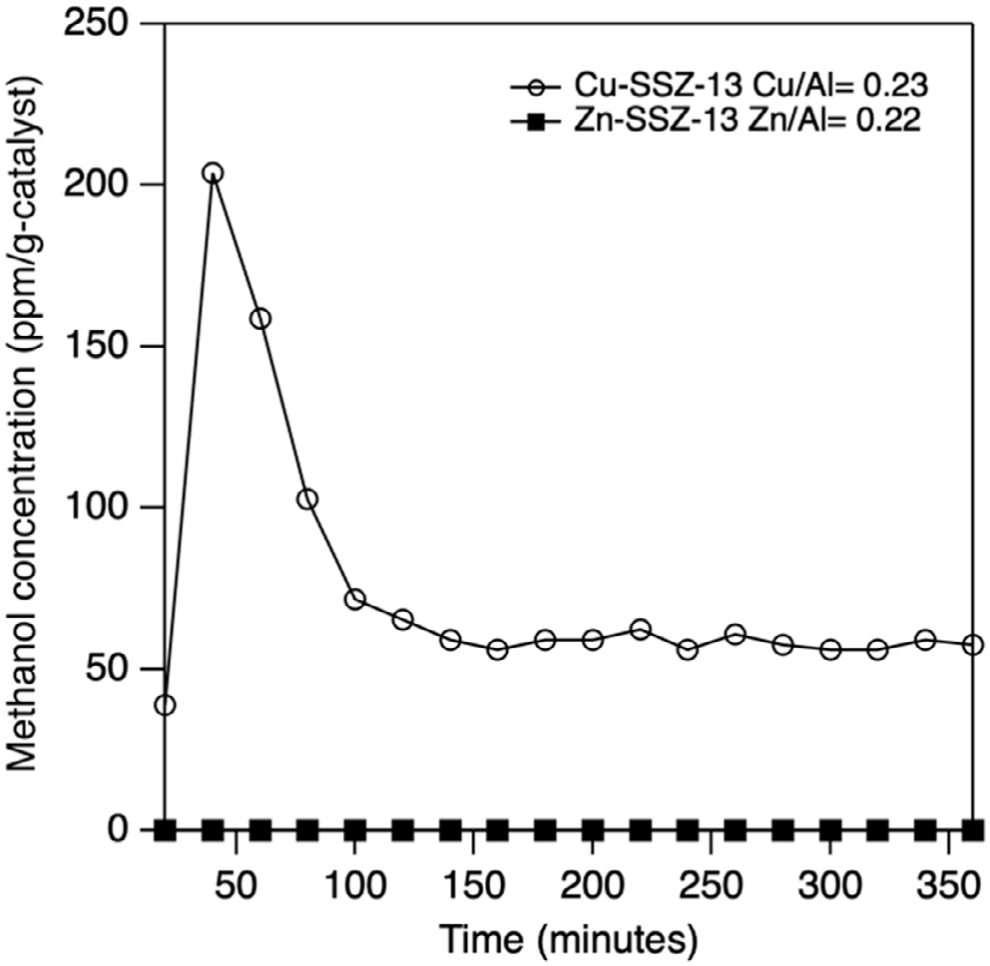
Plot of methanol effluent content versus time for Cu‐SSZ‐13 (Cu/Al = 0.23) and Zn‐SSZ‐13 (Zn/Al = 0.22).

**Figure 4:**
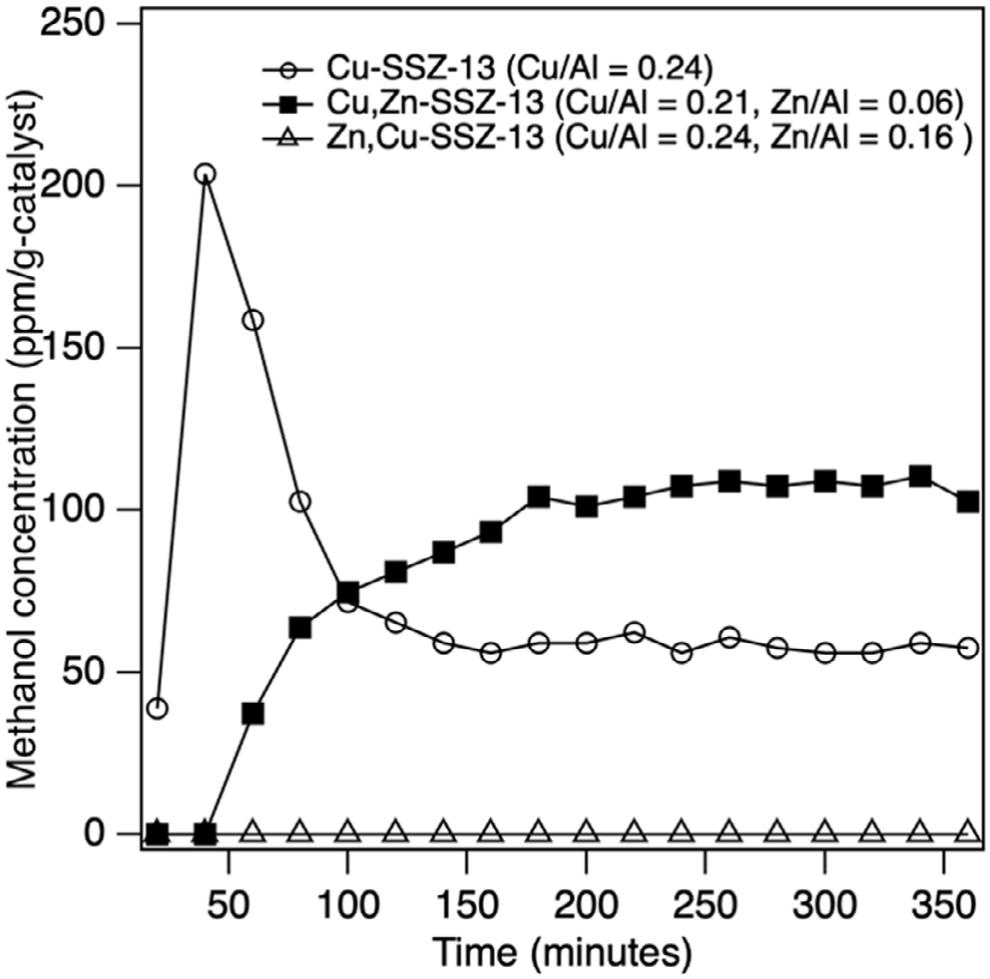
Plot of methanol effluent content versus time for Cu‐SSZ‐13 (Cu/Al = 0.24), Cu,Zn‐SSZ‐13 (Cu/Al = 0.21, Zn/Al = 0.06), and Zn,Cu‐SSZ‐13 (Cu/Al = 0.24, Zn/Al = 0.16).

The Cu‐SSZ‐13 (Cu/Al = 0.23) with medium copper loading was tested for the methane to methanol reaction. As seen in previous works in this research space,^[^ ^36^, ^38^, ^39^ ^]^ there is a spike in methanol production at the beginning of the reaction, typically from start to about 120 min, because of the preadsorbed methane from the dry run of methane for 40 min. After the first 100–200 min, the methanol production stabilizes at a STY of 14.5 ± 0.30 mmol mol‐Cu‐h^−1^ and SA of 6.1 ± 0.12 μmol g‐h^−1^ (**Figure** **3**). These results were comparable to results in prior literature^[^ ^38^ ^]^ (SA of 6.05 ± 0.40 μmol g‐h^−1^ and STY of 13.2 ± 0.9 mmol mol‐Cu‐h^−1^) with similar copper loading of 2.5 wt%. It should also be noted that methanol is the only product observed, i.e., no carbon dioxide is detected in the International Energy Agency (2024), World Energy Outlook 2024, IEA. Licence: Creative Commons Attribution CC BY‐NC‐SA 4.0 gas chromatograph mTCD. By contrast, Zn‐SSZ‐13 (Zn/Al = 0.22) with a similar metal loading was tested, and it did not produce any methanol. This confirms our initial hypothesis that Zn‐SSZ‐13 is not an active catalyst for this reaction. **Figure** **4** shows one set of samples with both copper and zinc directly compared to the pure copper sample.

There are a few conclusions to be drawn from this figure. Like Figure 3, the sample with the highest zinc content does not produce any methanol, even with copper present. By contrast, the Cu,Zn‐SSZ‐13 (Cu/Al = 0.21, Zn/Al = 0.06) sample produces more methanol than the pure copper sample. Again, analogous to the Cu‐SSZ‐13, we do not detect any carbon dioxide or other products. The Cu,Zn sample has a STY of 26.4 ± 0.42 mmol mol‐Cu‐h^−1^ and SA of 11.5 ± 0.18 μmol g‐h^−1^, giving an 82% increase in STY and 87% increase in SA compared to Cu‐SSZ‐13. This shows that small quantities of zinc can help to increase the methanol production of Cu‐SSZ‐13 catalysts.

**Figure 5:**
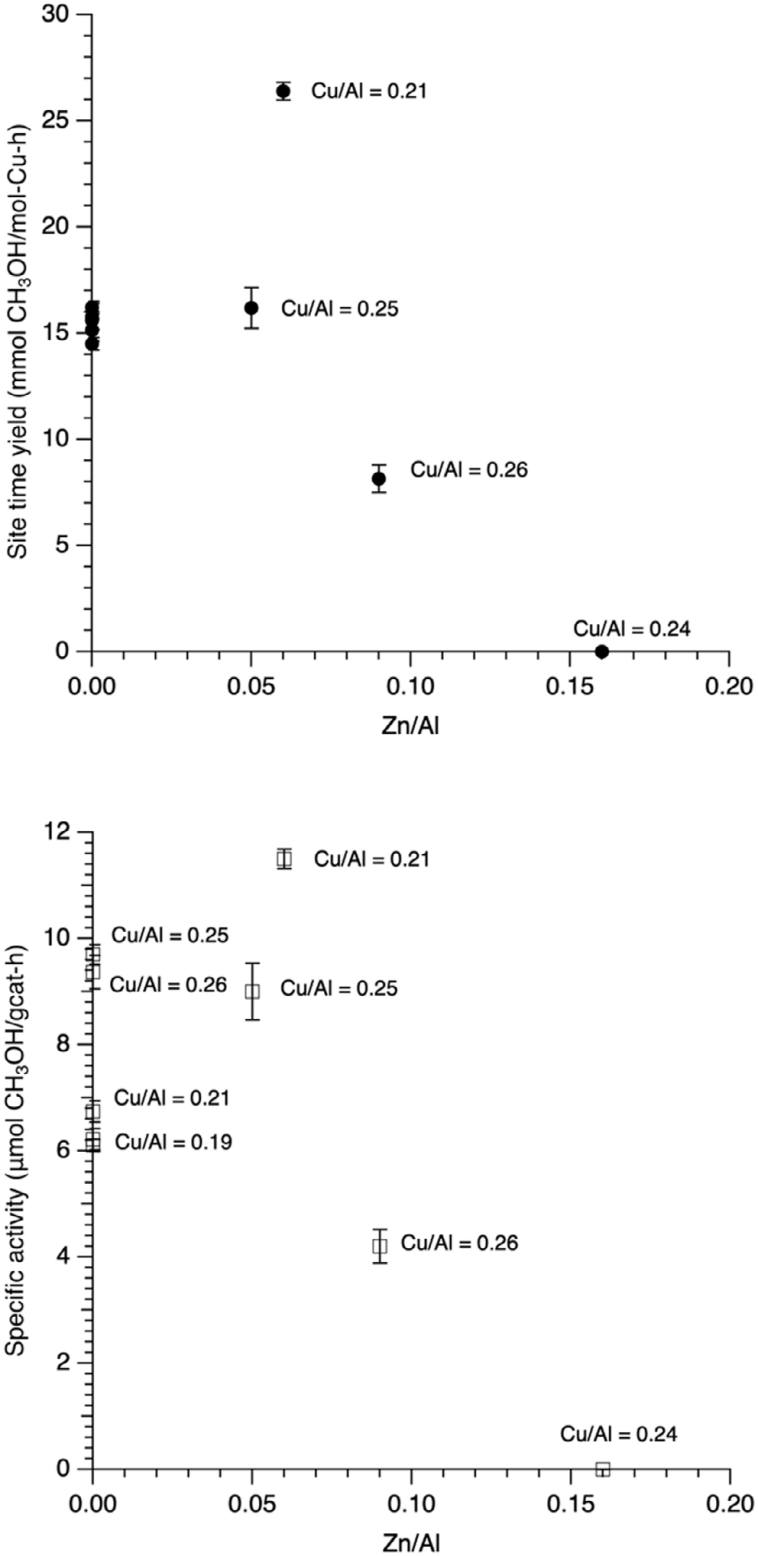
Plot of STY (top) and SA (bottom) versus Zn/Al for samples with copper loadings of between Cu/Al = 0.2 and 0.26. Points on the y‐axis (Zn/Al = 0) for the STY plot correspond to Cu/Al = 0.25, 0.21, 0.19, 0.24, and 0.26 from top to bottom, with errors between (+/−) 0.3 and 0.8.

To better understand possible reasons for this behavior, the next step was to make samples with approximately the same copper content (Cu/Al = 0.2–0.25) with varying levels of zinc. **Figure** **5** shows the STY (top) and SA (bottom) for a series of samples with what we will refer to as having moderate copper loading. In contrast to the results in Figure 4, at slightly higher copper loading (Cu/Al = 0.25+/‐0.01), we saw little to no difference between Cu‐SSZ‐13 (Cu/Al = 0.25) and Cu,Zn‐SSZ‐13 (Cu/Al = 0.25, Zn/Al = 0.05). Finally, with the last pair, Cu‐SSZ‐13 (Cu/Al = 0.26) and Cu,Zn‐SSZ‐13 (Cu/Al = 0.26, Zn/Al = 0.09), we saw a significant decrease in activity both in terms of STY and SA. The Cu/Zn ratio does not seem to be key in controlling activity. Rather, we believe that the total metal content (i.e., (Cu+Zn)/Al)) is important for determining reactivity at these higher copper contents. This is ascribed to the fact that as the M/Al increases above 0.3, there is a reduction in acid sites to the point where the rate is deleteriously impacted. This in fact is what is observed for samples that contain only copper at levels above Cu/Al of 0.3.^[^ ^43^ ^]^ We revisit this issue of the effect of the Cu/Zn ratio below in the context of samples that contain less copper.

### 3.2 Effect of Zinc Content (Cu/Al ≈ 0.1)

**Figure 6:**
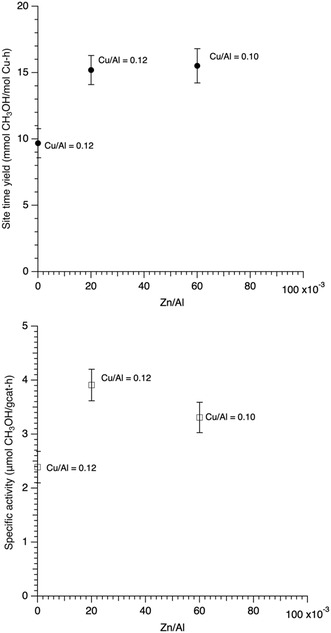
STY and SA for Cu‐SSZ‐13 and Cu,Zn‐SSZ‐13 samples with a Cu/Al of 0.1 (+/−0.02).

Given the results above, it was decided to prepare samples with a lower copper content, ≈Cu/Al = 0.1, to observe if zinc has the same effect as it did at higher Cu/Al values. The elemental analysis showed the parent Cu‐SSZ‐13 material had a Cu/Al = 0.12. Two additional samples were made from this parent material: a sample with Cu/Al = 0.12, Zn/Al = 0.02 (Zn/Cu = 0.167), and another with Cu/Al = 0.1, Zn/Al = 0.06 (Zn/Cu = 0.6). **Figure** **6** shows that the methanol production increased for both Cu,Zn‐SSZ‐13 samples as compared to the parent Cu‐SSZ‐13. The samples are ≈50% more active than the parent copper sample on a SA basis, and 50%–60% more active on a site time basis. It is also worth pointing out that the activity of these two samples is comparable to the Cu/Al = 0.2–0.25 samples without zinc. Interestingly, both Cu,Zn samples are, within the error bars, identical. So, here, the reactivity appears to be insensitive to the Cu/Zn ratio as samples with a Cu/Zn of six behave very similar catalytically to a sample with Cu/Zn of ≈1.6.

**Figure 7:**
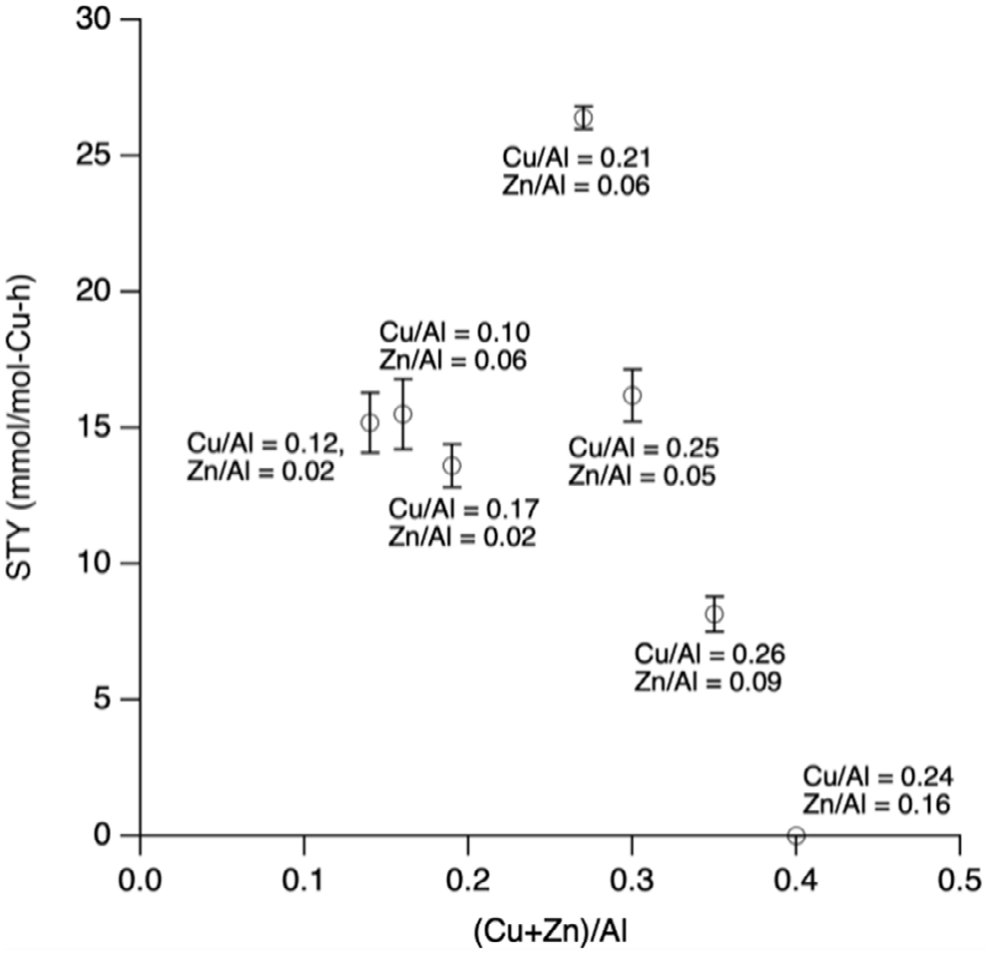
Plot of STY versus (Cu+Zn)/Al for samples in Figure 4, 5, 6 that contain both zinc and copper.

Another way to visualize this is shown in **Figure** **7**, where a plot of STY versus total metal content (Cu + Zn)/Al is plotted for all the Cu,Zn samples. The data in the figure shows that at copper loadings below 0.2, the STY is insensitive to the total metal content. However, one can see at copper loadings of 0.2 and above that STY systematically decreases with the total metal content. The key finding in this work is that at both low and medium copper loading, it has been demonstrated that the methanol production of Cu‐SSZ‐13 can be improved by exchanging a small amount of zinc into Cu‐SSZ‐13.

The total metal loading (M = (Cu + Zn))/Al appears to be governing activity once an M/Al of 0.3 or higher is reached. In contrast, this is not observed at lower M/Al values. Prior work has shown that the methanol production drops as the metal loading increases (M/Al > 0.3).^[^ ^39^ ^]^ This reiterates the point that Brønsted acid sites, which help to increase methanol formation, have to be present in the catalyst for methane to methanol conversion.^[^ ^45^, ^46^ ^]^

**Figure 8:**
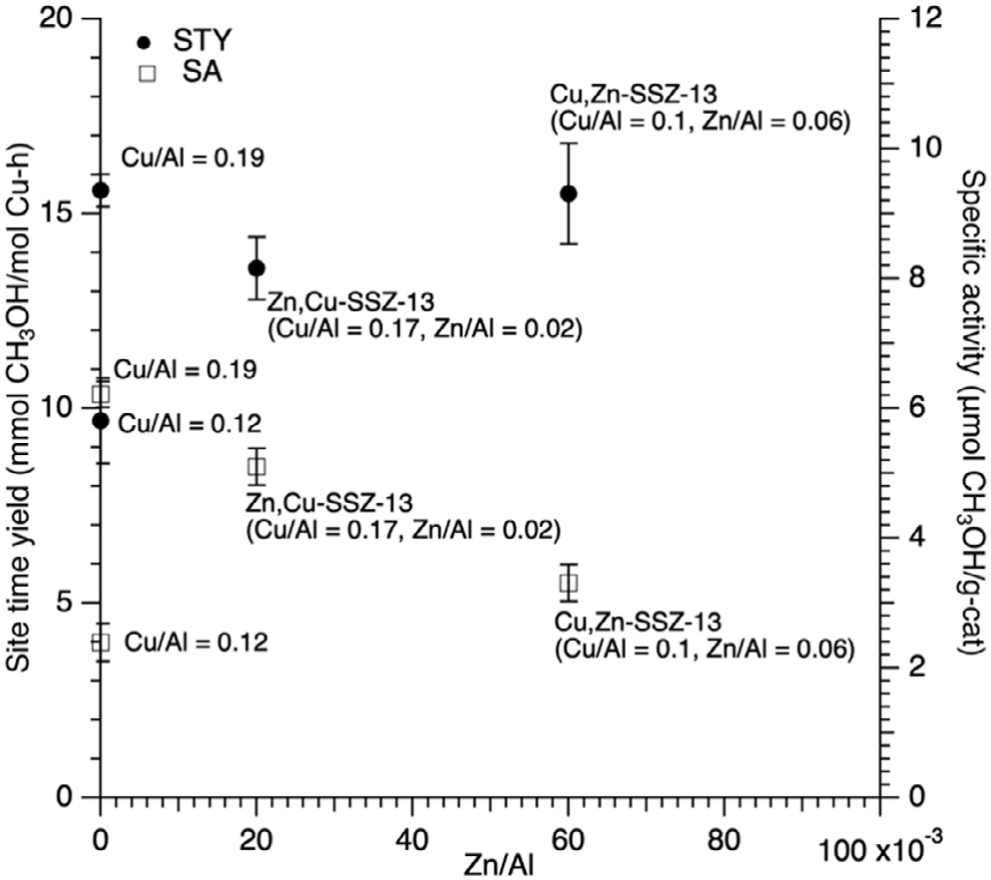
Plot of STY (left axis, filled circles) and SA (right axis, open squares) for Cu‐SSZ‐13 (Cu/Al = 0.12, and 0.19), Cu,Zn‐SSZ‐13 (Cu/Al = 0.1, Zn/Al = 0.06), and Zn,Cu‐SSZ‐13 (Cu/Al = 0.017, Zn/Al = 0.02).

An issue we were interested in exploring is whether the sequence of metal addition is important. To study that, we prepared a small number of samples, where Zn‐SSZ‐13 was first generated, and then this material was exchanged with copper. The first item of note is that in our hands, it was very difficult to load copper into Zn‐SSZ‐13 during the second step in an incremental way. Rather what is observed is that copper rapidly displaces 40%–60% of the zinc in the zeolite. This tends to lead to high total metal loading (Cu + Zn) in Zn,Cu‐SSZ‐13 samples. By contrast, it was much easier to incrementally load zinc into Cu‐SSZ‐13 as the zinc‐exchange step into Cu‐SSZ‐13 displaces a far smaller amount (≈10%) of the copper. **Figure** **8** shows a plot of STY and SA for Zn,Cu‐SSZ‐13 (Cu/Al = 0.17, Zn/Al = 0.01), two Cu‐SSZ‐13 samples (Cu/Al = 0.19, Cu/Al = 0.12), and Cu,Zn‐SSZ‐13 (Cu/Al = 0.10, Zn/Al = 0.06). One observation is that the Zn,Cu sample has a slightly lower STY and SA as compared to the pure copper sample with the same total metal content (Cu + Zn/Al = 0.19). Also of note is that the low copper content Cu,Zn sample (Cu/Al = 0.1, Zn/Al = 0.06) is more active than the Zn,Cu sample with similar total metal content.

### 3.3 Infrared Spectroscopy Studies

So, the question is, why does the addition of a small amount of zinc lead to noticeable increases in methanol production. Turning to the literature, studies have shown that the copper atoms in the eight‐membered ring (8MR) of the SSZ‐13 cage catalyze the reaction.^[^ ^53^ ^]^ Prior structural work on Cu‐SSZ‐13^[^ ^54^ ^]^ has clearly shown that in dehydrated Cu‐SSZ‐13, copper(II) prefers to initially populate the double six‐membered rings (D6MR). How this changes in the presence of polar molecules complicates this picture. One hypothesis, drawing from the S. An et al. work showed that zinc cations help to improve the dispersal of copper leading to increase in the proportion of copper in active sites for methane to methanol reaction when exchanged together into ZSM‐5 catalytic material^[^ ^55^ ^]^ and mordenite catalytic material,^[^ ^50^ ^]^ is that zinc prefers to sit more strongly into the double six‐membered rings than copper, and as a result the small number of zinc cations exchanged in result in the copper cations migrating into the eight‐membered ring sites.^[^ ^56^, ^57^ ^]^

**Figure 9:**
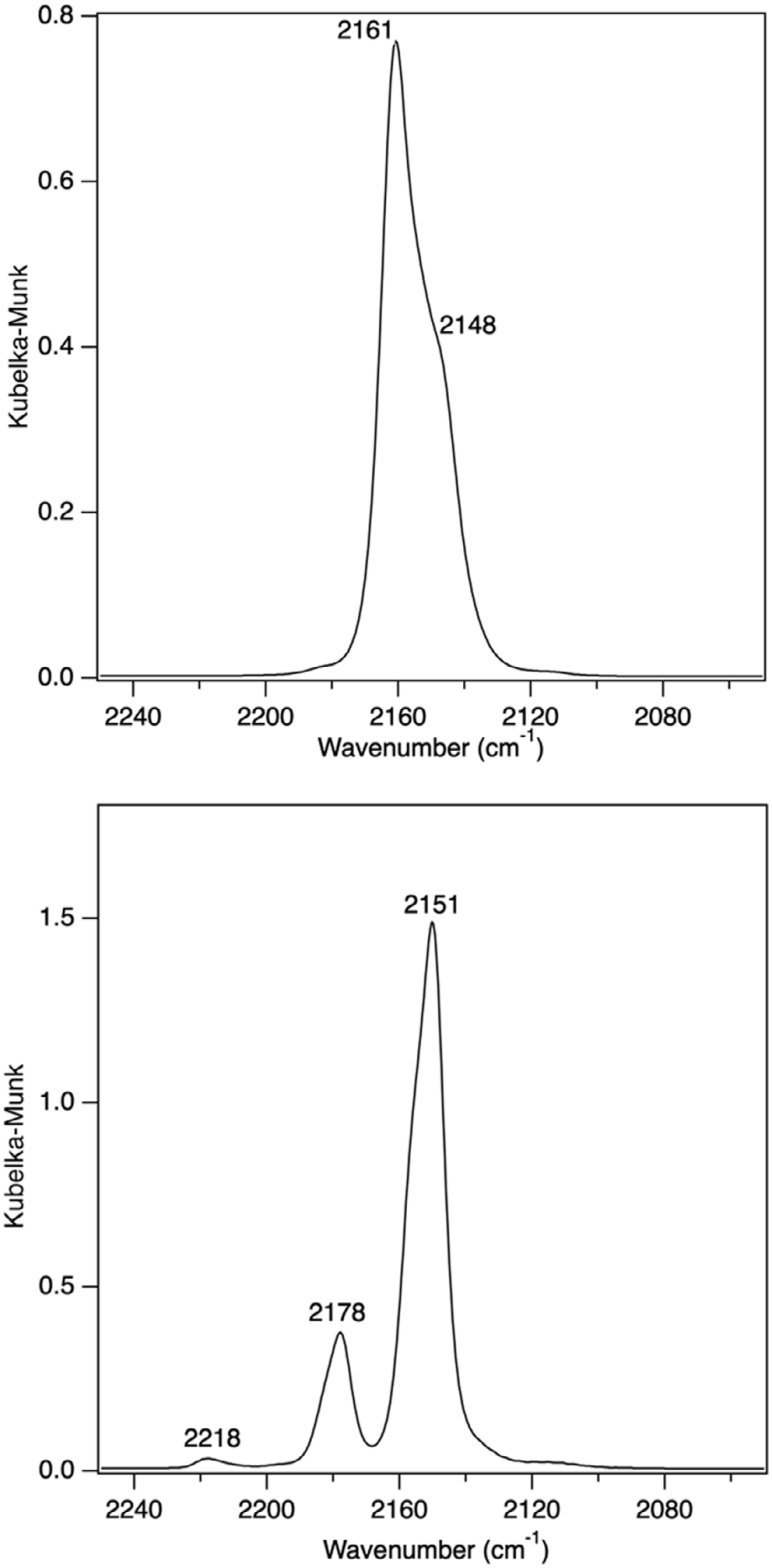
(Top) DRIFTS Spectra for Cu‐SSZ‐13 and (bottom) Cu,Zn‐SSZ‐13.

**Figure** **9** shows infra‐red spectroscopy results for dehydrated samples of Cu‐SSZ‐13 (Cu/Al = 0.23) and our ‘champion catalyst’ (Cu/Al = 0.21, Zn/Al = 0.06) after exposure to CO. The pure copper samples show two features, one at 2148 cm^−1^ and one at 2161 cm^−1^. Previous work has shown that the CO feature at 2148 cm^−1^ is due to CO in a more constrained environment (e.g., double six‐membered ring), whereas the feature at 2161 cm^−1^ is associated with CO bound to Cu(I) in eight‐membered rings. The CO results for the Cu,Zn sample show a very interesting result in that the features are shifted. The feature at 2148 cm^−1^ is now at 2151 cm^−1^, and the 2161 cm^−1^ is now dramatically shifted to 2178 cm^−1^. The feature at 2218 cm^−1^ is attributed to CO associated with zinc cations based on the pure Zn‐SSZ‐13 samples showing only one feature at this wavenumber (Figure S3, Supporting Information). The explanation for this shift in the 2161 cm^−1^ feature is that the copper in the presence of a small amount of zinc becomes more electron‐deficient. Electron‐deficient copper has been shown in different work to result in improved catalytic activity, stability, and selectivity in different direct redox reactions such as ammonia abatement,^[^ ^58^ ^]^ ammonia production,^[^ ^59^ ^]^ and other applications.^[^ ^60^, ^61^ ^]^ This could be the origin of the enhanced reactivity.

## 4 Conclusion

We have been able to demonstrate that zinc can be exchanged into Cu‐SSZ‐13, and the amount of zinc exchanged into the Cu‐SSZ‐13 can be tuned using exchange temperature and exchange time. Cu,Zn‐SSZ‐13 can lead to higher methanol production than a corresponding Cu‐SSZ‐13 with similar copper loading. This is observed at both moderate (Cu/Al = 0.2) and lower (Cu/Al = 0.1) copper loadings. Samples containing only zinc are catalytically inactive. By contrast, small levels of zinc (Zn/Al = 0.06 or less) lead to a marked increase in methanol production. In the best case, Cu,Zn‐SSZ‐13 (Cu/Al = 0.21, Zn/Al = 0.06) has a STY of 26.4 ± 0.42 mmol mol‐Cu‐h^−1^ and SA of 11.5 ± 0.18 μmol g‐h^−1^. This is over an 80% increase in both STY and SA over Cu‐SSZ‐13 samples with comparable copper loadings (Cu/Al = 0.2–0.26). A similar, but slightly less modest improvement, is observed for Cu,Zn samples at a copper loading of ≈0.12, where 50% increases in methanol production over just copper samples are observed. In the case of the lower copper content, the addition of zinc results in a material that is as active as the best pure copper SSZ‐13 samples. Infrared spectroscopy results suggest that the presence of zinc makes the copper more electron‐deficient, providing one possible explanation for the increased activity. This work provides further evidence of the utility of multiple metals in zeolites, and more specifically, another path to pursue to increase methanol production from methane over metal‐containing zeolites.

## Conflict of Interest

The authors declare no conflict of interest.

## Supporting information

Supplementary Material

## Data Availability

The data that support the findings of this study are available from the corresponding author upon reasonable request.
