# Supplementary material for: Low Levels of Zinc Exchanged into Cu‐SSZ‐13 Increase Methanol Production in the Partial Oxidation of Methane to Methanol
Source: ChemistryOpen. 2025 Jul 29;14(12):e202500352. doi: 10.1002/open.202500352 (PMC12680563; doi:10.1002/open.202500352)
Supplement: Supplementary file 1 — Supplementary Material [file OPEN-14-e202500352-s001.pdf]

Supporting Information: Low levels of Zinc Exchange into Cu-SSZ-13 Increases Methanol Production in the Partial Oxidation of Methane to Methanol

Motunrayo Ogunleye,[a] Hridita Purba Saha,[b] Ayman M. Karim,[b] and Daniel F. Shantz*[a]

[a]Department of Chemical and Biomolecular Engineering, Tulane University, 6823 St. Charles Avenue, New Orleans, LA 70118, United States.

[b]Department of Chemical Engineering, Virginia Polytechnic Institute and State University, Blacksburg, VA 24061, United States.

Email: dshantz@tulane.edu

Ion Exchange protocol for the Cu,Zn-SSZ-13 (Cu/Al=0.21, Zn/Al=0.06) sample

Cu-SSZ-13 exchange: Copper-exchanged SSZ-13 (Cu-SSZ-13) samples were prepared by ion exchange of 1g of NH_4-SSZ-13 with 200 mL of a 0.05M copper (II) sulfate solution at room temperature for 1 h. The product was recovered by vacuum filtration and washed with deionized water. The wet solids were placed in the oven at 80°C overnight to dry. Elemental composition analysis was found to be Cu/Al = 0.220

Cu,Zn-SSZ-13 exchange. Copper, zinc exchanged SSZ-13(Cu,Zn-SSZ-13) samples were prepared by mixing 1g of Cu-SSZ-13 with 100mL of 0.05M $ZnSO_4$, depending on the copper loading, at 80 °C for 30 minutes to obtain Cu,Zn-SSZ-13 samples. The wet solids (Cu,Zn-SSZ-13) were placed in the oven at 80°C overnight until dry. Elemental composition analysis was found to be Cu/Al = 0.207 Zn/Al = 0.058

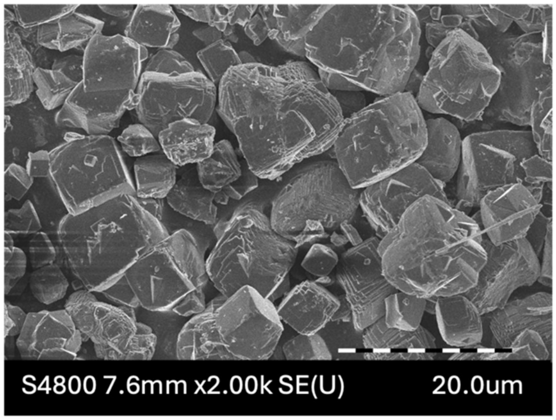

(a) Calcined SSZ-13

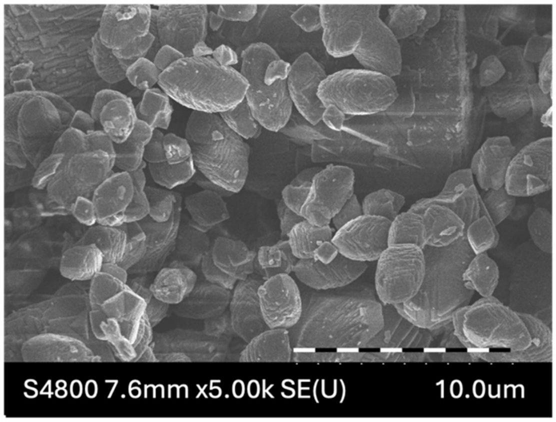

(b) Cu-SSZ-13

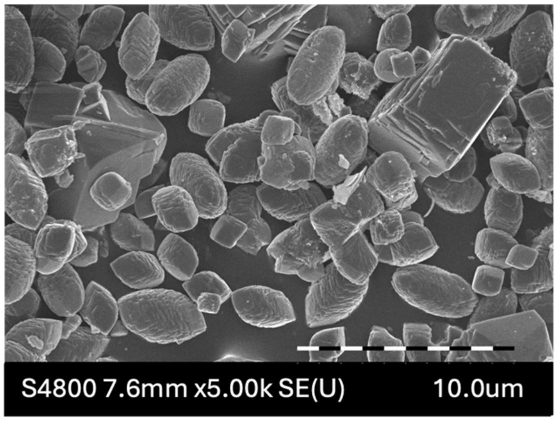

(c) Zn-SSZ-13

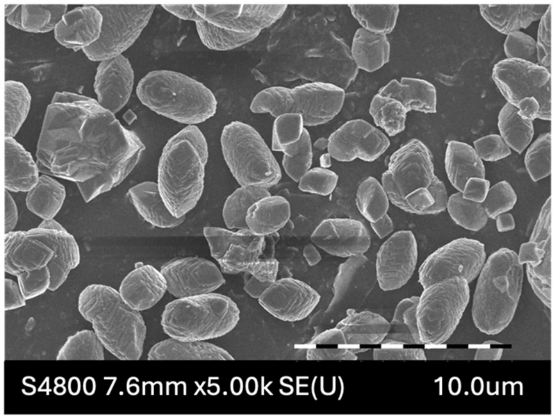

(d) Cu,Zn-SSZ-13

Figure S1. SEM image of (a) SSZ-13 (b) Cu-SSZ-13 (c) Zn-SSZ-13 (d) Cu,Zn-SSZ-13.

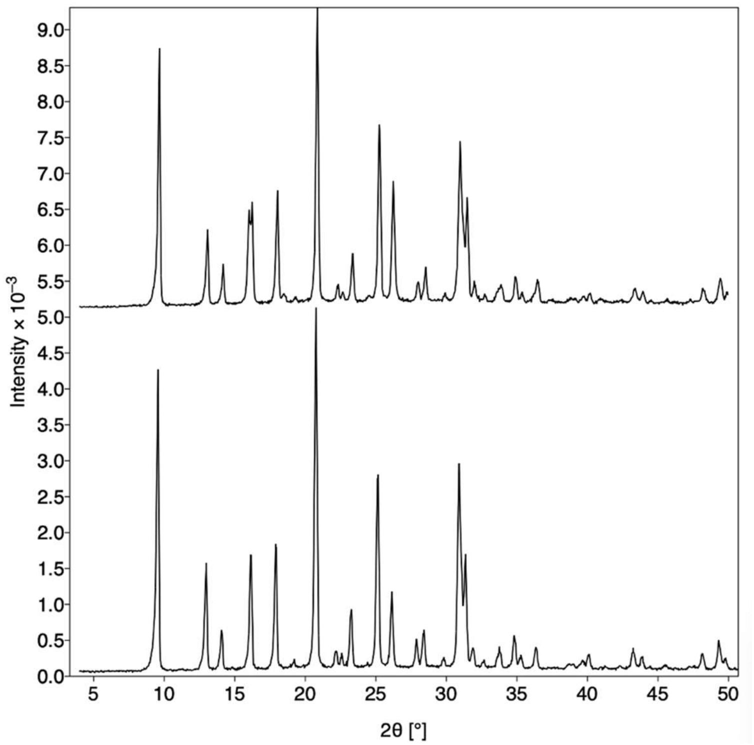

Figure S2. XRD of Cu,Zn-SSZ-13 samples before and after use as a catalyst in methane to methanol reactor showing no changes in the XRD pattern.

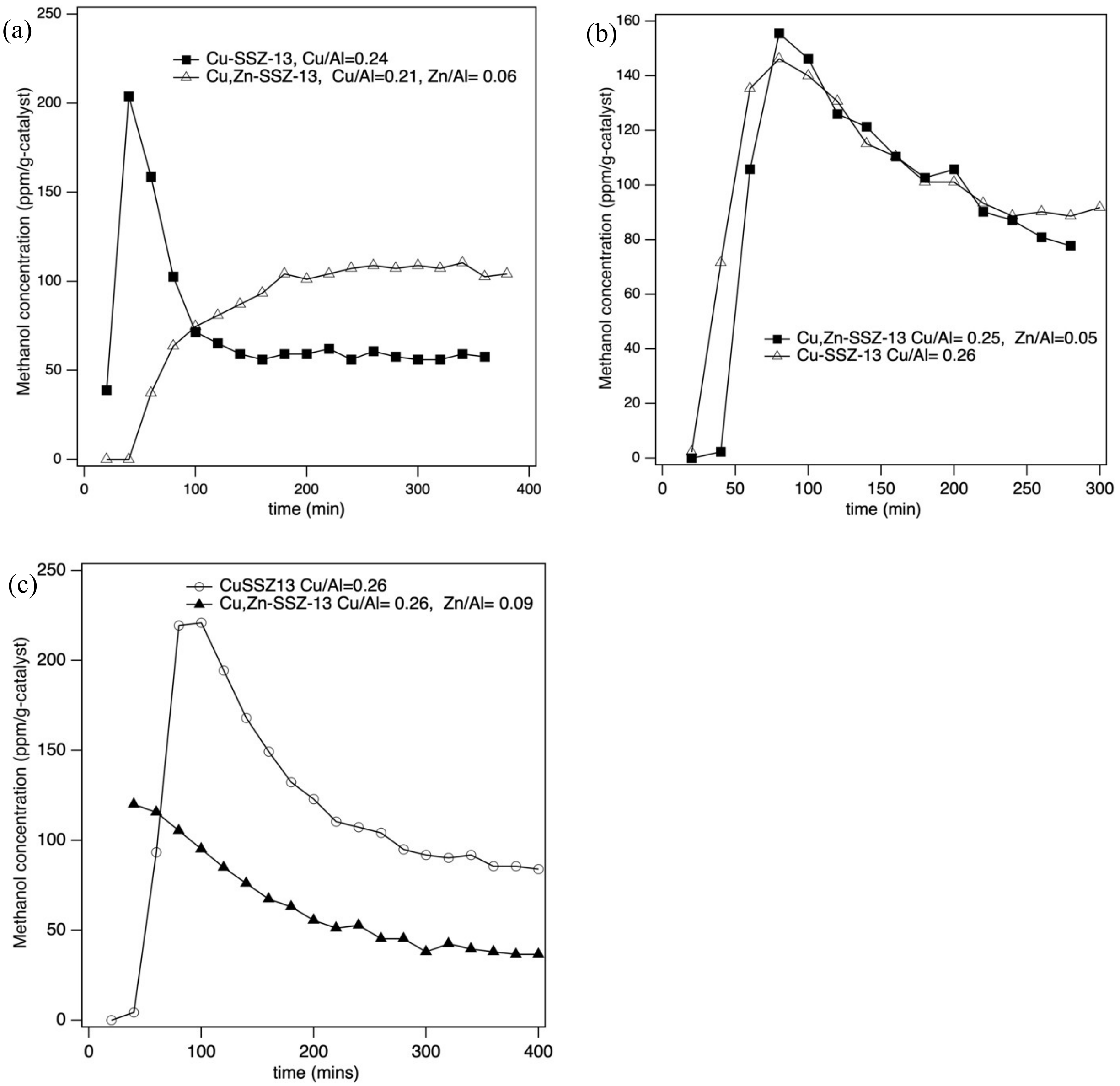

Figure S3. Methanol production over Cu,Zn-SSZ-13 with Cu/Al ≈ 0.25. (a) Cu-SSZ-13 (Cu/Al = 0.24), and Cu,Zn-SSZ-13 (Cu/Al = 0.21, Zn/Al = 0.06). (b) Cu-SSZ-13 (Cu/Al = 0.26), and Cu,Zn-SSZ-13 (Cu/Al = 0.25, Zn/Al = 0.05). (c) Cu-SSZ-13 (Cu/Al = 0.26), and Cu,Zn-SSZ-13 (Cu/Al = 0.26, Zn/Al = 0.09).

Exchange	Initial M/Al	Volume/g zeolite	Conc.	Metal salt	Temp.	Time	Zn/Al	Cu/Al
**Cu into NH_4-SSZ-13**	0	200 ml/g	0.05M	Copper sulfate	Room temp.	1 hour	0	0.2201
**Zn into NH_4-SSZ-13**	0	100 ml/g	0.05M	Zinc sulfate	Room temp.	1 hour	0.034	0
**Zn into Cu-SSZ-13**	0.2201	200 ml/g	0.05M	Zinc nitrate	80°C	30 minutes	0.058	0.207
**Cu into Zn-SSZ-13**	0.034	200ml/g	0.05M	Copper sulfate	Room temp.	1 hour	0.02	0.165

Table S1. Elemental composition of samples using varying metal salt, temperature and volume resulting in different metal loading.

Sample	Site Time Yield (mmol/mol-Cu-h)	Specific Activity (μmol/g-h)
Cu/Al = 0.21	15.77 ± 0.62	6.74 ± 0.27
Cu/Al = 0.21, Zn/Al = 0.06	26.4± 0.4	11.46 ± 0.18
Cu/Al = 0.24, Zn/Al = 0.16	0	0
Cu/Al = 0.17, Zn/Al = 0.02	13.6 ± 0.75	5.1 ± 0.28
Cu/Al = 0.21	15.8 ± 0.62	6.7 ± 0.27
Cu/Al = 0.19	15.6 ± 0.42	6.2 ± 0.17
Cu/Al = 0.124	9.7 ± 1.1	2.4 ± 0.29
Cu/Al = 0.1, Zn/Al = 0.06	15.5 ± 1.29	3.31 ± 0.28
Cu/Al = 0.12, Zn/Al = 0.02	15.2 ± 1.10	3.91 ± 0.29
Cu/Al = 0.25	16.2 ± 0.3	9.7 ± 0.18
Cu/Al = 0.25, Zn/Al = 0.05	16.18± 0.95	9.0± 0.53
Cu/Al = 0.26	15.14± 0.52	9.37± 0.32
Cu/Al = 0.26, Zn = 0.09	8.15± 0.64	4.2± 0.32

Table S2. Site time yields and Specific activity values for samples shown in Figures 5-9.

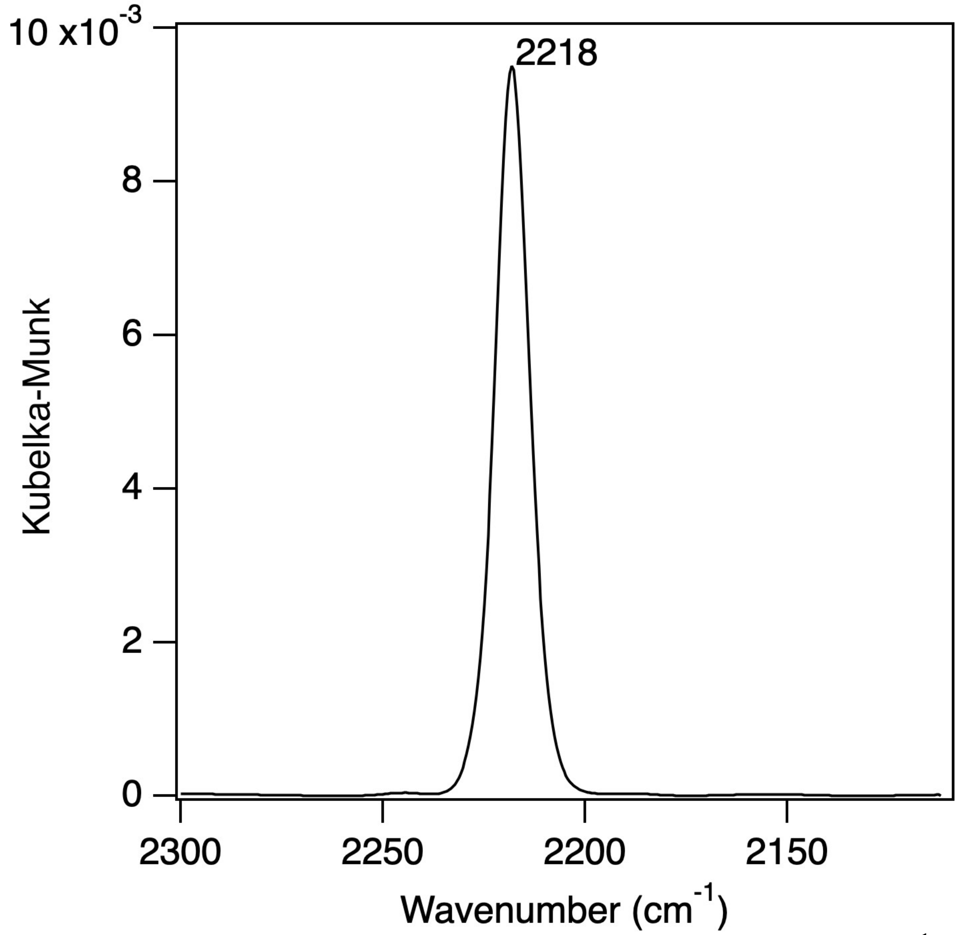

Figure S3. DRIFTS Spectrum for Zn-SSZ-13 showing peak at 2218cm^{-1}.
